# Rapid Etiological Classification of Meningitis by NMR Spectroscopy Based on Metabolite Profiles and Host Response

**DOI:** 10.1371/journal.pone.0005328

**Published:** 2009-04-24

**Authors:** Uwe Himmelreich, Richard Malik, Till Kühn, Heide-Marie Daniel, Ray L. Somorjai, Brion Dolenko, Tania C. Sorrell

**Affiliations:** 1 Centre for Infectious Diseases and Microbiology and Westmead Millennium Institute and NHMRC Centre of Clinical Research Excellence in Infections and Bioethics in Haematological Malignancies, The University of Sydney, Westmead, Australia; 2 Institute for Magnetic Resonance Research, The University of Sydney, St Leonards, New South Wales, Australia; 3 Post Graduate Foundation in Veterinary Science and Faculty of Veterinarian Sciences, The University of Sydney, St Leonards, New South Wales, Australia; 4 Bruker Biospin, Fällanden, Switzerland; 5 Unité de Microbiologie, Mycothèque de l'Université catholique de Louvain, Louvain-la-Neuve, Belgium; 6 Institute for Biodiagnostics, National Research Council of Canada, Winnipeg, Manitoba, Canada; The Research Institute for Children at Children's Hospital New Orleans, United States of America

## Abstract

Bacterial meningitis is an acute disease with high mortality that is reduced by early treatment. Identification of the causative microorganism by culture is sensitive but slow. Large volumes of cerebrospinal fluid (CSF) are required to maximise sensitivity and establish a provisional diagnosis.

We have utilised nuclear magnetic resonance (NMR) spectroscopy to rapidly characterise the biochemical profile of CSF from normal rats and animals with pneumococcal or cryptococcal meningitis. Use of a miniaturised capillary NMR system overcame limitations caused by small CSF volumes and low metabolite concentrations. The analysis of the complex NMR spectroscopic data by a supervised statistical classification strategy included major, minor and unidentified metabolites.

Reproducible spectral profiles were generated within less than three minutes, and revealed differences in the relative amounts of glucose, lactate, citrate, amino acid residues, acetate and polyols in the three groups. Contributions from microbial metabolism and inflammatory cells were evident. The computerised statistical classification strategy is based on both major metabolites and minor, partially unidentified metabolites. This data analysis proved highly specific for diagnosis (100% specificity in the final validation set), provided those with visible blood contamination were excluded from analysis; 6–8% of samples were classified as indeterminate.

This proof of principle study suggests that a rapid etiologic diagnosis of meningitis is possible without prior culture. The method can be fully automated and avoids delays due to processing and selective identification of specific pathogens that are inherent in DNA-based techniques.

## Introduction

Bacterial meningitis is an acute disease with a high mortality [1], [2]. Severe neurological sequelae have been reported in 25% of cases [3], [4]. Outcomes are directly related to the speed with which the diagnosis is established and therapy initiated. Conventional diagnosis relies on screening examination of cerebrospinal fluid (CSF) for non-specific markers such as inflammatory cells, proteins and glucose, and the more specific but insensitive Gram stain for micro-organisms, to distinguish rapidly between bacterial and non-bacterial (most commonly viral and fungal) infection. Ultimate confirmation of the diagnosis and identification of the specific microbial pathogen by culture is sensitive and specific, but slow. This might delay the most appropriate treatment. Relatively large volumes of CSF are recommended to maximise sensitivity and provide sufficient material for completion of the various diagnostic tests.

Alternative approaches based on chemicals in CSF due to production by the infection causing microorganism or due to the immune response of the host are potentially useful for the diagnosis of a variety of neurological diseases, including meningitis [5]. Metabolic changes that reflect infection such as low glucose or disproportionately elevated lactate are characteristic of bacterial meningitis but have been poor discriminators in some studies [6]–[8]. A disadvantage of these biochemical methods is that only particular compounds are targeted by individual tests. High-throughput technologies such as Nuclear Magnetic Resonance (NMR), Infrared (IR) and Raman spectroscopy, chromatographic methods and mass spectrometry, generate complex data (“fingerprints”) based on chemical composition and metabolite profiles of micro-organisms (metabolome) [9]–[13]. Such metabolomic methods can detect genotypic and phenotypic differences even in closely related microorganisms and also in genetically modified strains [13]–[17], with greater discriminatory power than transcriptomics and proteomics [17], [18]. Spectroscopic techniques characterise rapidly and simultaneously multiple chemical compounds in biological fluids [10], [19]–[28]. In addition, NMR spectroscopy detects low molecular weight metabolites, requires no time-consuming sample preparation and is non-destructive.

The metabolite composition of normal CSF has been studied intensively by NMR spectroscopy [22], [29], [30]. CSF contains a relatively limited repertoire of metabolites, compared with serum or urine, that are relatively stable with diet and medication, and on storage at room temperature for a short time [22], [29], [31]. CSF is therefore a potentially useful biofluid for identifying metabolic profiles of pathogens and the host response.

Computerised methods utilize the whole NMR spectrum and include unnoticed (or unknown) compounds. In a pilot study of acute meningitis, CSF spectra from small numbers of controls, patients with viral meningitis and bacterial/fungal meningitis were distinguished using an unsupervised cluster analysis method [8], suggesting that classification according to etiology is possible using larger data sets. Since human cases of meningitis caused by different bacterial or fungal species are relatively uncommon in developed countries and acquisition of sufficient spectral data would require several years, a proof of principle study was performed using rat models of meningitis. Repeated sampling of small volumes of CSF from individual animals was made possible by the use of a miniaturised micro NMR system [32], [33].

In clinical practice, rapid identification of a specific bacterial or fungal pathogen is important as it enables immediate initiation of appropriate antimicrobial therapy based on predictable patterns of antibiotic susceptibility and hence improves clinical outcomes. We have utilised a metabolomic approach based on NMR spectra from CSF of animal models to evaluate if metabolites produced by an infection-causing microorganism or a specific immune response can be utilised for diagnosis. This could provide the basis for further development of the method on human CSF specimens. The two disease models used were meningitis caused by *Cryptococcus neoformans* and *Streptococcus pneumoniae*.

## Results

### Meningitis models and metabolite profiles

Meningitis was induced in Fisher 344 rats by injection of *Cryptococcus neoformans* (N = 31) or *Streptococcus pneumoniae* (N = 30) into the cisterna magna. CSF samples were collected from these animals after first signs of meningitis and at several time points thereafter. Control CSF samples were repeatedly collected from sham injected animals (phosphate buffered saline (PBS), N = 5) or prior infection.

**Figure 1:**
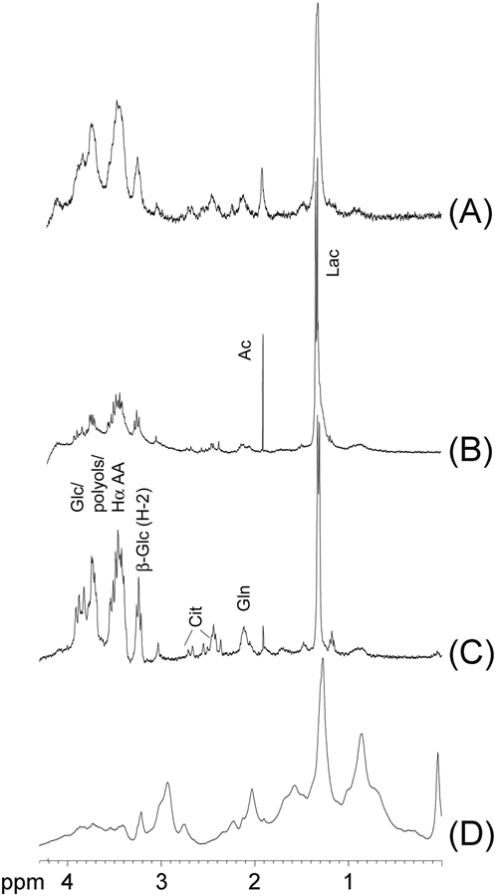
^1^H NMR spectra acquired with a 400 MHz spectrometer equipped with probe for 1 mm sample tubes. NMR spectra were acquired from the following samples: (A) CSF from a control animal (before initiation of the infection), (B) CSF from an animal five days after injection of *S. pneumoniae* (animal showed symptoms of meningitis; heavy growth of *S. pneumoniae* from CSF confirmed streptococcal meningitis), (C) CSF from an animal four days after injection of *C. neoformans* (animal showed symptoms of meningitis, heavy growth of *C. neoformans* from CSF confirmed cryptococcal meningitis), (D) CSF heavily contaminated with blood (same animal as C but collected three days after initiation of the infection). Abbreviations refer to ^1^H NMR signal of the following metabolites: Ac – acetate, Ala – alanine, Cit – citrate, β-glc (H-2) – H-2 resonance of β-glucose, Glc – glucose, Gln – glutamine, Hα AA – resonances of H-α from amino acid residues, Lac – lactate.

Metabolite composition of the CSF samples was studied by NMR spectroscopy using either an NMR system equipped with an 1 mm micro probe or a conventional 5 mm probe. Figure 1 shows typical 1D NMR spectra of CSF samples from control animals (injected with PBS) and collected from animals with histologically confirmed meningitis, four days after injection of 10^4^ cfu of *C. neoformans* and *S. pneumoniae*, respectively. Resonances in the 1D NMR spectra from each pathology were assigned to respective metabolites using 2D NMR correlation spectra from at least five independent samples per category (see supporting material, Figure S1).

Although the major metabolites in CSF samples from all three categories were qualitatively similar for control and post-infection CSF samples from individual animals, quantitative differences were noted (supporting material, Table S1). CSF from infected animals contained increased amounts of lactate (5–90%, using the resonance at 1.31 ppm), decreased glucose (5–30%, resonance at 3.25 ppm) and marginally increased citrate (0–15% resonance at 2.45–2.75 ppm) and amino acid residues (0–15% for alanine (1.49 ppm) and glutamine/glutamate (2.2–2.4 ppm)). For most samples, only glutamine but no glutamate was detected. Some infected CSF samples showed increased acetate (for 55% of *S. pneumoniae* infections and for 30% of *C. neoformans* infections) and polyol signals (mannitol and glycerol resonances (3.90–3.95 ppm) for 20% of *C. neoformans* infections).

Integral ratios of the most abundant metabolites relative to glucose are summarised in Table S1. The quantitative changes that occurred in individual animals during the course of infection overlapped with variations in samples collected from different animals of the same class (control, *S. pneumoniae* and *C. neoformans*, see supporting material, Table S1), precluding the use of metabolite ratios for diagnosis of streptococcal and cryptococcal meningitis.

### Statistical classification of NMR data

A statistical classification strategy (SCS) [42] (an earlier version of which was previously used for metabolomic studies in pathogenic yeasts [16]) was utilised to improve predictability of class assignment based on NMR spectra, and also to identify metabolite differences occurring during pathogenesis in the meningitis models. A genetic-algorithm-based optimal region selection (GA-ORS) algorithm was used to identify discriminatory regions in the NMR spectra [34]. The three most discriminatory spectral regions identified by the GA-ORS for each pair-wise comparison (*S. pneumoniae* versus *C. neoformans*, *S. pneumoniae* versus control and *C. neoformans* versus control) are summarised in Table 1 (B). Metabolites within these spectral regions that were identified by 2D NMR techniques include the amino acid residues glutamine (traces of glutamate were found in 10% of the CSF samples independent of the pathology), valine, leucine, isoleucine; acetate, lactate, citrate, polyols and carbohydrate residues and α-hydroxybutyrate. Notably, polyols (mannitol/glycerol) were determined to contribute to the distinction between *C. neoformans* and *S. pneumoniae* meningitis but not to that between *C. neoformans* meningitis and controls, presumably because polyols overlap with glucose resonances, and glucose levels are higher in normal CSF than in that from animals with cryptococcal meningitis.

**Table 1:** Statistical Classification Strategy using NMR spectra of CSF samples.

(A) SCS analysis with and without blood contaminated CSF samples.			
	N	correct [%]	crisp [%]
**1. Training set (all incl.)**			
Control	52	97	73
*C.neoformans*	25	91	88
*S.pneumoniae*	24	95	88
**1. Validation set (all incl.)**			
Control	6	50	67
*C.neoformans*	15	64	93
*S.pneumoniae*	15	100	87
**Final Training set (excl. blood contaminations)**			
Control	49	96	94
*C.neoformans*	34	100	94
*S.pneumoniae*	39	97	92
**Final Validation set (excl. blood contaminations)**			
Control	12	100	92
*C.neoformans*	6	100	100
*S.pneumoniae*	6	100	84
**Inclusion of an additional clinical isolate**			
*C.neoformans* (WM1128)	5	80	100
*S.pneumoniae* (99-235-2193)	5	100	80

(B) Spectral regions used for the final SCS-based classifiers and potential metabolites of these regions that were also identified in 2D NMR spectra.			
	Method	Regions [ppm]	Potential metabolites
*C. neoformans* vs. *S. pneumoniae*	Rank-ordered 1^st^ derivatives	3.91–3.95	polyols (mannitol/glycerol) and carbohydrate residues
		2.66–2.70	citrate
		2.25–2.27	glutamine
*C. neoformans* vs. control	1^st^ derivatives	2.25–2.31	glutamine
		1.89–1.92	acetate, glutamine
		0.90–0.95	α-hydroxybutyrate, valine, leucine, isoleucine
*S. pneumoniae* vs. control	Rank-ordered 1^st^ derivatives	1.84–1.92	acetate, glutamine
		1.15–1.29	lactate
		0.77–0.88	valine, leucine, isoleucine

After identification of the most discriminatory regions in the NMR spectra of CSF, these regions were utilised to distinguish between the two pathologies and the uninfected rats. The final classification accuracy was achieved after repeated re-development and validation of the pair-wise classifiers. The first set of classifiers were based on all CSF samples (N = 101) and was still relatively inaccurate (Table 1). Although 91–97% of the spectra were assigned correctly to one of the classes, 12–27% of these assignments were ‘fuzzy’ (e.g., with low assignment confidence). These classifiers performed poorly on an independent validation sample set. It was noted that a high percentage of those spectra that were fuzzy or incorrect, were visibly contaminated with blood. Classifier redevelopment following exclusion of samples contaminated with blood resulted in final assignments with only 6–8% of samples classified fuzzy and 4% or less as incorrect (misclassification). Testing against a newly acquired, independent validation set (N = 34) from animals that were not part of the data set used for classifier development, resulted in 8–16% unreliable classification and 100% correct class assignment (Table 1). Animals were infected with an additional clinical isolate of *C. neoformans* (WM1128, n = 5) or *S. pneumoniae* (99-235-2193, n = 5). Both new isolates were classified mostly correctly (one isolate was misclassified and one was fuzzily assigned).

Repeated CSF collections from the same infected animal (N = 27) were mainly used for classifier validation. The time of collection of CSF after onset of meningitis did not influence class assignment. One possible explanation is that quantitative changes in metabolite composition between day 4 and 11 after infection in an individual animal were similar to differences in CSF composition between different animals collected at the same time point.

## Discussion

Micro-NMR spectroscopy of CSF when analysed by a supervised classification method distinguished rapidly and reproducibly between controls, rats with pneumococcal meningitis and rats with cryptococcal meningitis. This proof of principle study in experimental rats indicates that the etiology of meningitis can potentially be established rapidly without prior culture of the infection-causing microorganism using a very small amount of CSF. If able to be translated into clinical practice and extended to other pathogens, this is potentially a major diagnostic advance that would assist in the rapid selection of appropriate antimicrobial therapy and hence improve patient outcomes.

**Figure 2:**
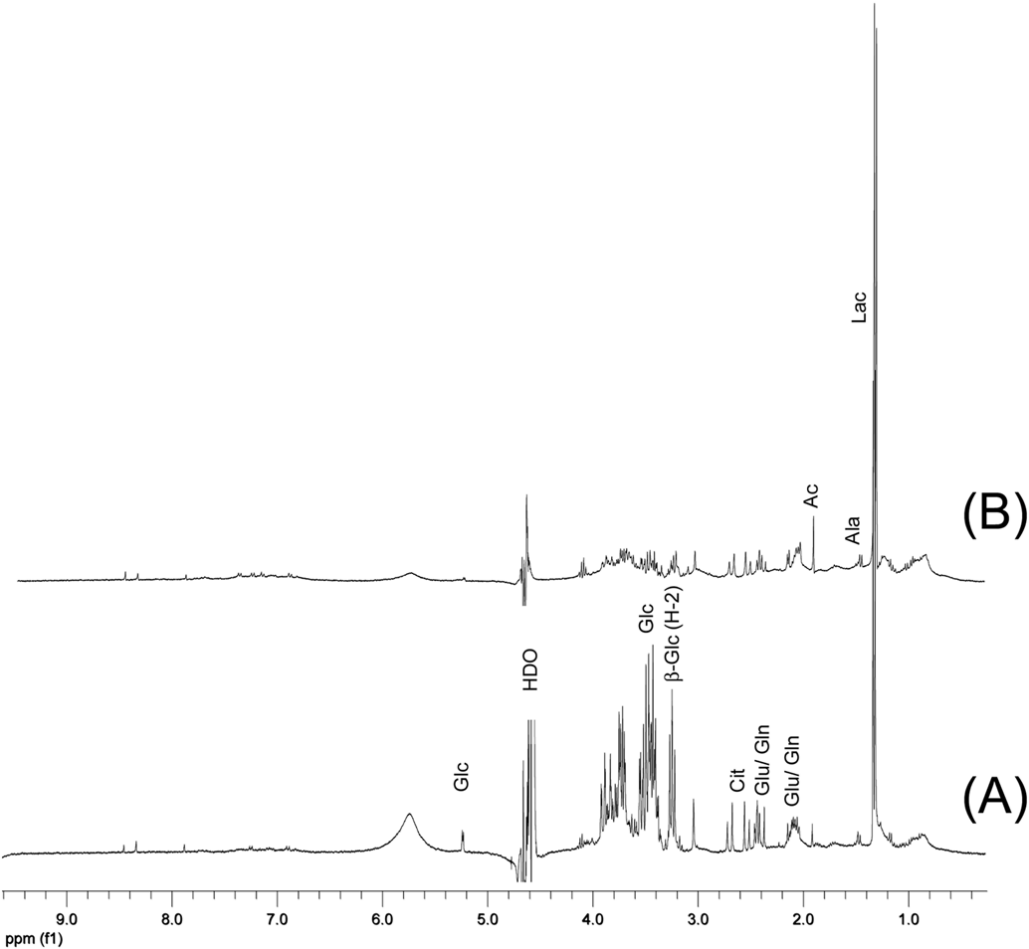
^1^H NMR spectra of 100 µl CSF samples from (A) a healthy rat and (B) a human not suffering from meningitis or other infections. The spectra acquired at 360 MHz using a susceptibility-matched 5 mm NMR tube. Abbreviations refer to ^1^H NMR signal of the following metabolites: Ac – acetate, Ala – alanine, β-glc (H-2) – H-2 resonance of β-glucose, Cit – citrate, HDO – remaining, partly deuterated water resonance, Glc – glucose, Gln – glutamine, Lac – lactate.

Human CSF is rich in NMR detectable metabolites. More than hundred have been identified based on NMR spectroscopy in CSF from patients with various neurological, metabolic and other non-neurological diseases [29], the most abundant being glucose and lactate, with lesser amounts of acetate, citrate, formate, 3-hydroxyl-butyrate, alanine, valine and glutamine [30]. The major metabolites in CSF samples from humans without infections or other apparent diseases are similar to those in healthy rats as observed in the present study (Figure 2). Rat models have previously proven to be suitable for NMR spectroscopic studies of staphylococcal and cryptococcal brain abscesses [35], [36].

Semi-quantitative analysis revealed that elevation of lactate and reduction in glucose levels were the dominant CSF metabolite changes in rats with meningitis. These changes are also documented in human cases of bacterial and fungal meningitis, but are not specific [8]. Changes in metabolite concentrations in CSF samples are due to a combined effect of metabolites from infecting microorganisms, inflammatory cells and changes due to the effect of meningitis on brain cell metabolism. For example, increased lactate levels in CSF may result from anaerobic cerebral metabolism (glycolysis) which occurs in response to brain cell ischemia and is a feature of both infectious and non-infectious neurological disorders [7], [37] but it is also a dominant metabolite of some bacteria under those conditions [38]. Notably, lactate is a prominent end product of metabolism in *S. pneumoniae* and levels in pneumococcal meningitis exceed those produced in individual human cases of staphylococcal and cryptococcal meningitis [8], suggesting a contribution from the organisms themselves. Further evidence of low concentrations of microbial metabolites in the CSF comes from mannitol identified in 20% of the CSF samples from animals infected with *C. neoformans* [39]. Low levels of glucose in bacterial meningitis might be due to reduced membrane carrier-facilitated glucose transport, microbial metabolism and increased cerebral glycolysis secondary to ischemia and cytokine release [7], but are also seen in subarachnoid hemorrhage [37].

Although some metabolic differences were detected by analysing subjectively chosen spectral regions, discrimination between the two pathologies and controls would not have been possible if based solely on these major metabolites. In the present study, the selection of the most discriminatory regions of the NMR spectra by GA-ORS suggests that glutamine (2.25–2.27/2.3 ppm) may have contributed most to the distinction between the three pathologies (control CSF, cryptococcal and pneumococcal meningitis specimen), which was in principle confirmed by the comparison of individual integral regions (supporting material, Table S1). However, other unidentified minor metabolites may have been more discriminatory, underlining the advantages of spectral analysis by a statistical classification strategy compared to assignment of individual, preselected key metabolites. Elevated glutamine, acetate and the amino acids valine, leucine and/or isoleucine as well as polyols may have contributed to the classification between pneumococcal or cryptococcal meningitis and the respective controls as indicated in the spectral regions of Table 1. For example, increased glutamine has been observed in bacterial meningitis [40] and acute subarachnoid haemorrhage [37] and reflects decreased neuronal activity.

Differences in the relative abundance of recognisable metabolites, exemplified by the integral ratios of lactate, acetate, glutamine, citrate and polyols relative to glucose in the present study, were insufficient for classification based on changes in relative concentrations of those individual compounds. Although the analysis of NMR spectra by GA-ORS identified most discriminatory regions that might indicate certain key metabolites, the complex nature of the spectra with composite resonances makes it difficult to identify all, even minor contributors to the successful classification. Such low concentration metabolites are most likely not recognised by operator-based assignment of the simultaneously detected resonances. This is particularly true for the spectral region between 3.5 to 4.0 ppm where many metabolites resonate, including those of importance for classification (for example carbohydrates, polyols like mannitol and all Hα resonances of amino acid residues). Those metabolites can often only be identified by time consuming 2D correlation spectra (see supporting material, Figure S1).

### Technical considerations

Accurate classification was achieved in the present study without any pre-processing of the CSF samples. As also reported by Maillot [29], contamination of CSF with blood did influence classification accuracy and resulted in exclusion of 10% of samples. Blood contamination is a potentially greater problem with repeated cisternal taps in small animals than it is with a single spinal fluid collection in humans.

A desirable precondition for the incorporation of NMR spectroscopy into clinical diagnostics is automation of sample handling and data analysis, and reduction of the sample volume to a minimum. Even more important is the utilization of small volumes for repeated monitoring in animal models. We therefore chose a 1 mm micro-NMR probe for acquisition of NMR spectra. Low volumes of CSF samples (5 to 35 µl) were directly collected into 1 mm micro NMR tubes. Subsequent snap-freezing and later, automated acquisition of NMR spectra resulted in a satisfactory signal-to-noise ratio and spectral quality. Comparison between data acquisition using the 1 mm micro NMR probe with those in conventional 5 mm tubes confirmed previous findings of dramatically improved signal-to-noise ratios and equal or better spectral resolution and water suppression by using 1 mm micro tubes [33]. No additional sample preparation was performed apart from addition of deuterated water (D_2_O) to some samples (N = 30) for evaluation of spectral quality (resolution) after shimming. No significant differences were found between these samples and sample acquisition performed without shimming. Thus the micro-volume NMR system represents a powerful advance. The stability of CSF samples at room temperature for some hours makes the combination of the NMR system with automatic sample changers feasible, allowing rapid, high-throughput data acquisition and analysis.

### Concluding points

We have demonstrated for *C. neoformans* and *S. pneumoniae* infections that diagnosis of meningitis according to etiology of the infection causing microorganisms is possible without prior culture of the microorganism using a metabolomics based approach. The method can be fully automated and is suitable for incorporation in clinical diagnosis. This would potentially accelerate and hence greatly improve turn-around-times compared with current diagnostic procedures once it has been extended to other meningitis-causing pathogens and validated on clinical samples.

## Materials and Methods

### Ethics statement

Animal experimentation was carried out according to the Australian National Health and Medical Research Council Guidelines and with ethical approval from the University of Sydney Animal Ethics Committee (approval number K14/12-97/3/2668).

Clinical samples were studied in compliance with ethical approval granted by the Human Ethics Review Committee of Sydney Western Area Health Service.

### Animal studies

A total number of 175 CSF samples were collected from 76 animals (Fisher 344 rat strain, 150–250 g, Animal Research Council, Perth, WA, Australia) in this study. Rats were anaesthetized by inhalation of 2% halothane (May & Baker, Degenham, UK) in oxygen delivered from a precision out-of-circuit vaporiser using a tight-fitting facemask. Following induction of deep anaesthesia, a 25-gauge needle was inserted into the cisterna magna and 15–35 µl of CSF was removed by suctioning the hub of the needle using an insulin syringe. These CSF samples were used as control (sterile) specimens. Meningitis was induced by the slow injection of 15–35 µl PBS containing 10^4^ cfu of clinical isolates of *Cryptococcus neoformans* (isolate WM628, 31 rats infected) or *Streptococcus pneumoniae* (isolate 99-241-1187, 30 rats infected), followed by rapid withdraw of the needle. Sham injection was performed for five animals by injection of sterile PBS. For validation of the classification five animals per pathogen were injected using an additional clinical isolates of *C. neoformans* (isolate WM1128) and *S. pneumoniae* (isolate 99-235-2193).

CSF samples (15–35 µl) from infected rats were harvested when animals first showed signs of meningitis (such as head tilt, seizures, loss of appetite; typically four to eight days following inoculation) or after day eight if the animals remained asymptomatic. Additional collection of CSF was performed three days thereafter for all animals that had not been euthanized (N = 27). CSF was collected as before from the cisterna magna. Part of the CSF sample from all animals was used for confirmation of the presence of microorganisms using standard microbiological tests (10–15 µl CSF). Only CSF samples from which the respective microorganisms had been cultured were included in the NMR study. The presence of cryptococcal organisms in CSF was confirmed by thin smears of CSF (10 µl), which were stained with a rapid Romanowsky stain (DiffQuik; Lab Aids, Australia). Typically, large numbers of organisms were evident in stained smears. Plate counts were performed on CSF samples from thirteen animals (six *C. neoformans* and seven *S. pneumoniae*) by culture of five different dilutions on horse blood agar plates for 24 hours (*S. pneumoniae*) and on Sabouraud dextrose agar plates for 48 hours (*C. neoformans*) at 35°C. Three inoculated animals were excluded from the study due to an absence of microorganisms in CSF samples.

Animals were given free access to food and water *ad libitum* and maintained in a 12 hours light/dark cycle at 25°C throughout the study. Animals were monitored daily for any signs of distress. Rats were sacrificed after the experiments. Euthanasia was generally performed with CO_2_.

Surplus CSF from clinical samples was studied in compliance with ethical approval granted by the Human Ethics Review Committee of Sydney Western Area Health Service. CSF from patients with cryptococcal meningitis were stored at −80°C for up to three months before NMR analysis. The diagnosis of cryptococcal meningitis was made independently by attending clinicians and reviewed by TCS for consistent clinical features. Leukocyte counts, biochemistry, microbial strains and cultures were performed for all samples. Cryptococcal meningitis was defined by a positive India Ink stain and/or CSF cryptococcal antigen titre >8 and/or culture of *C. neoformans* from CSF.

### NMR studies

Five to fifteen microliters of CSF were directly collected into micro NMR tubes (1 mm outer diameter, 0.8 mm inner diameter, Bruker BioSpin AG, Fällanden, Switzerland). The samples were transferred within 15 minutes into liquid nitrogen and stored at −70°C for up to 60 days for NMR experiments. Samples were carefully thawed before NMR experiments and centrifuged for 30 seconds using a manual centrifuge to avoid air bubbles in the micro tubes. Deuterated water (2–4 µl) was added to 30 samples to adjust for low filling heights.

NMR experiments were carried out as described [33]. In brief, 1D ^1^H NMR spectra for SCS analysis were acquired using a Bruker Biospin Avance 400 spectrometer. Two-dimensional (2D) correlation NMR spectra for resonance assignment were acquired using a Bruker Biospin Avance 600 spectrometer. An appropriate TXI 1 mm MicroProbe with z-gradients was used on either NMR spectrometer. In addition, NMR spectra were also acquired from CSF samples collected at the terminal time point (volume 50–150 µl) using a Bruker Biospin Avance 600 spectrometer equipped with a 5 mm {^1^H, ^13^C} inverse-detection dual-frequency probe. All measurements were carried out without spinning at 37°C. 1D ^1^H NMR spectra were acquired with 16 k data points, a spectral width of 10 ppm, a repetition time of three seconds and accumulation of 64 averages. Residual water was suppressed using an 1D NOESY presaturation sequence with a mixing time of 100 ms [41]. For the suppression of contributions from macromolecules and other compounds with short T2 values, the Carr-Purcell-Meiboom-Gill (CPMG) experiment was performed on a small set of CSF samples (see supporting material Table S2). Experimental parameters were: number of repeated cycles 200, echo time 10 ms.

#### Resonance assignment

2D homo- and heteronuclear correlation spectra were acquired for six to eight CSF samples per class to assign ^1^H NMR resonances to respective metabolites (see supporting material, Figure S1). Standard {^1^H, ^1^H} COSY and {^1^H, ^1^H} TOCSY experiments were acquired with the following parameters: spectral width in t_2_ 10 ppm, t_2_ time domain 2 K, 256 increments of 8 or 16 acquisitions each, relaxation delay 1 s. TOCSY spectra with mixing times of 40 ms were acquired with 256 increments of 2 K data points and 16 acquisitions. Standard sensitivity-enhanced gradient inverse-detection HSQC spectra were acquired with the following parameters: optimisation for one-bond coupling of 125 and 145 Hz, total of 256 increments with 32 transients, 4 K complex data points, and ^13^C decoupling using GARP-1. HMBC spectra were optimised for one-bond coupling of 125 Hz and long range coupling constants of 6 Hz.

Relative quantification of metabolites was achieved by integration of resonances in the chemical shift region between 0.0–4.0 ppm, following polynomial baseline correction.

### Statistical Classification Strategy

^1^H NMR spectra of CSF from controls and animals with confirmed *S. pneumoniae* and *C. neoformans* meningitis (obtained three to eight days after infection) were used to develop three pair-wise classifiers (for *S. pneumoniae* versus *C. neoformans; C. neoformans* versus control and *S. pneumoniae* versus control) as described previously [16], [42], [43]. In brief: magnitude NMR spectra were normalized to the total integral between 0.35 to 4.0 ppm, which contains 1500 data points. Two to three maximally discriminatory regions of these spectra were identified by a genetic-algorithm-based Optimal Region Selector (GA-ORS) [34]. These regions are summarised in Table 1. Using the first derivatives or the rank ordered first derivatives of the spectral regions, pair-wise Linear Discriminant Analysis based classifiers were developed. The robustness of the LDA classifiers was tested using a bootstrap-based crossvalidation by randomly selecting half of the spectra to develop the classifiers and the remaining half to validate the classifiers [44]. This process was repeated 1000 times with random replacements. The classifiers yielded probabilities of class assignments for the individual spectra. Class assignment was called crisp if class assignment probabilities were >66%. Software developed in-house was used for all steps of the statistical classification (IBD, NRC Canada, Winnipeg) as described before [34], [42], [43].

## Supporting Information

Figure S1 (0.34 MB DOC)

Table S1 (0.03 MB DOC)

Table S2 (0.03 MB DOC)
