# Supplementary material for: Rapid Etiological Classification of Meningitis by NMR Spectroscopy Based on Metabolite Profiles and Host Response
Source: PLoS One. 2009 Apr 24;4(4):e5328. doi: 10.1371/journal.pone.0005328 (PMC2669500; doi:10.1371/journal.pone.0005328)

# Supporting Material:

**Figure S1:** One- and two-dimensional NMR spectra to illustrate part of the resonance assignments for the CSF samples. NMR spectra were collected from a CSF sample of a rat with confirmed cryptococcal meningitis. The spectra show (A) 1D ^1^H NMR spectrum, (B) {^1^H, ^1^H} COSY spectrum, (C) 2D J-resolved NMR spectrum, (D) {^1^H, ^13^C} HSQC spectrum (optimized for ^1^J=145Hz) and (E) {^1^H, ^13^C} HMBC spectrum (optimized for ^1^J=125Hz and ^n^J=6Hz). Abbreviations refer to the following metabolites: AA Hα - α resonances of amino acid residues, Ac – acetate, Ala – alanine, Cit – citrate, Glc –glucose, Gln – glutamine, HDO – remaining, partly deuterated water resonance, Lac – lactate, Lys – lysine, Man – mannitol. Numbers and greek letters refer to the position of the respective H- and C-atoms. Note: Only metabolites that were mentioned in the text and were considered of importance are labeled.


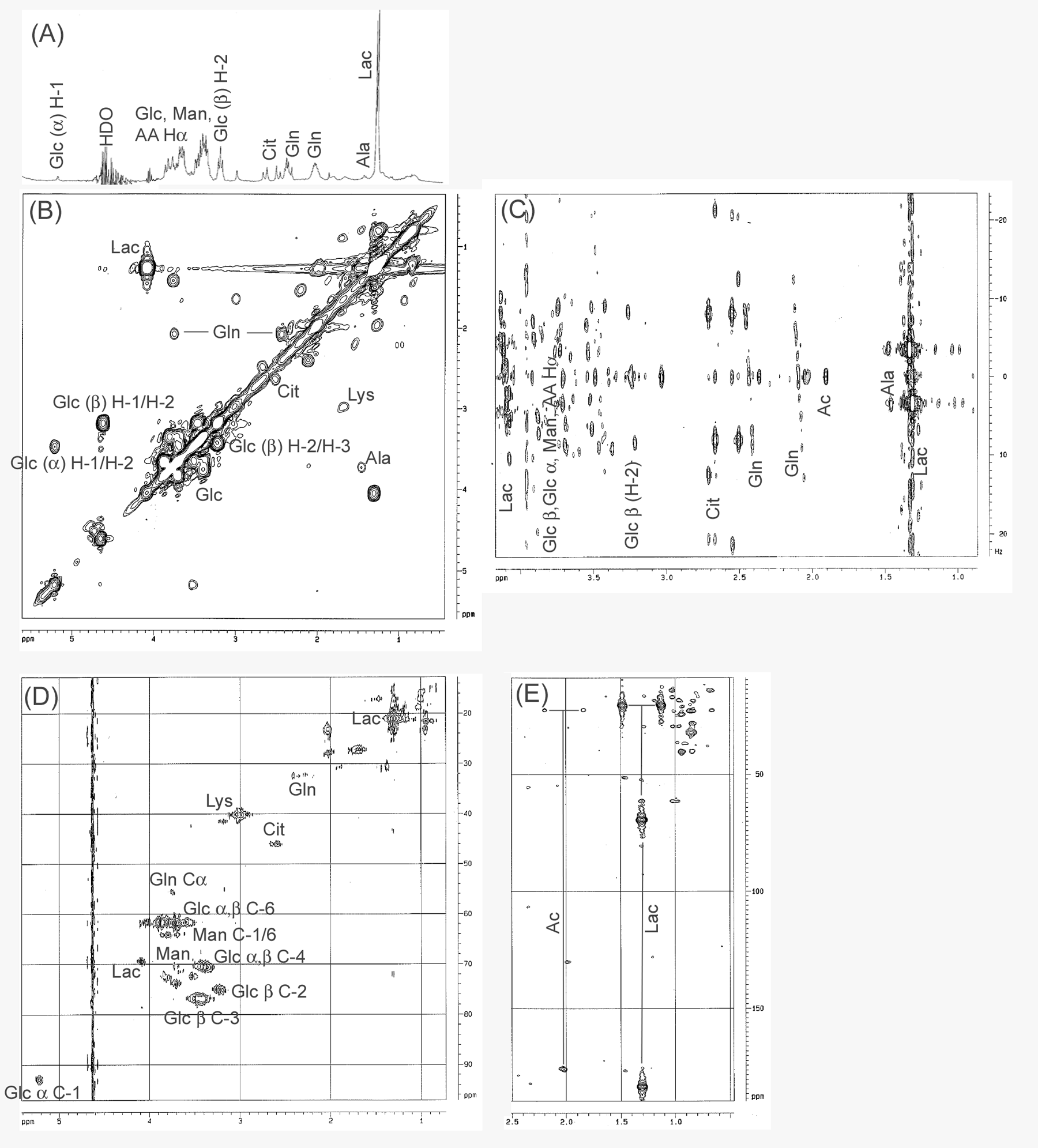

Supplement: Figure S1 — (0.34 MB DOC) [file pone.0005328.s001.doc]
