# Supplementary material for: Rapid Etiological Classification of Meningitis by NMR Spectroscopy Based on Metabolite Profiles and Host Response
Source: PLoS One. 2009 Apr 24;4(4):e5328. doi: 10.1371/journal.pone.0005328 (PMC2669500; doi:10.1371/journal.pone.0005328)
Supplement: Table S1 — (0.03 MB DOC) [file pone.0005328.s002.doc]

# Supporting Material:

# Table S1: Comparison of metabolite ratios. NMR spectra from CSF samples used for the training process of the SCS classifiers were quantitatively analysed. The range of the integral ratios is shown for the classes control (C, N=49), *S. pneumoniae* meningitis (SP, N=39) and *C. neoformans* meningitis (CN, N=34). Integral ratios were determined after phase and baseline correction. The listed main metabolites represent compounds that were identified by 2D correlation NMR spectra. However, other metabolites may also have contributed to the respective signals. Metabolite ratios were expressed relative to the H-2 resonance of β-glucose due to its presence in all samples and its clear separation from other resonances. No adjustment for the number of represented protons was performed. The resonance frequencies represent the center of the integral region (width 0.05ppm).

| Resonance ratio | Main metabolites | C | SP | CN |
| --- | --- | --- | --- | --- |
| 1.31 : 3.25 ppm | Lactate : Glucose (H-2α) | 5.4-7.2 | 5.9-9.1 | 6.2-8.7 |
| 1.91 : 3.25 ppm | Acetate : Glucose (H-2α) | nd-0.01 | nd-0.02 | nd-0.02 |
| 2.20 : 3.25 ppm | Glutamine^$^ : Glucose (H-2α) | 0.1-0.4 | 0.2-0.5 | 0.1-0.6 |
| 2.72 : 3.25 ppm | Citrate : Glucose (H-2α) | 0.05-0.1 | 0.05-0.2 | 0.05-0.2 |
| 3.75 : 3.25 ppm | Mannitol, Glycerol, Glucose (H-6) : Glucose (H-2α) | 1.9-3.2 | 1.7-3.1 | 2.1-3.7 |

nd-not detectable.

^$^ The signal mainly comprised of glutamine. However, small amounts of glutamate were detectable but not quantifiable in some samples.
