# Supplementary material for: Rapid Etiological Classification of Meningitis by NMR Spectroscopy Based on Metabolite Profiles and Host Response
Source: PLoS One. 2009 Apr 24;4(4):e5328. doi: 10.1371/journal.pone.0005328 (PMC2669500; doi:10.1371/journal.pone.0005328)
Supplement: Table S2 — (0.03 MB DOC) [file pone.0005328.s003.doc]

# Supporting Material:

# Table S2: Comparison of metabolite ratios using CPMG experiments. In order to suppress contributions from macromolecules that may potentially mask small concentrations of metabolites, CPMG NMR spectra were acquired. Comparison of metabolite ratios with those from standard ^1^H NMR spectra (Table S1) did not show significant differences for a small data set from CSF of controls (C, N=15), *S. pneumoniae* meningitis (SP, N=12) and *C. neoformans* meningitis (CN, N=12). Integral ratios were determined after phase and baseline correction. No adjustment for the number of represented protons was performed. The resonance frequencies represent the center of the integral region (width 0.05ppm).

| Resonance ratio | Main metabolites | C | SP | CN |
| --- | --- | --- | --- | --- |
| 1.31 : 3.25 ppm | Lactate : Glucose (H-2α) | 5.0-6.9 | 5.8-9.5 | 5.7-8.2 |
| 1.91 : 3.25 ppm | Acetate : Glucose (H-2α) | 0.01-0.05 | 0.01-0.03 | 0.02-0.05 |
| 2.20 : 3.25 ppm | Glutamine : Glucose (H-2α) | 0.1-0.3 | 0.2-0.4 | 0.2-0.4 |
| 2.72 : 3.25 ppm | Citrate : Glucose (H-2α) | 0.08-0.2 | 0.08-0.3 | 0.06-0.2 |
| 3.75 : 3.25 ppm | Mannitol, Glycerol, Glucose (H-6) : Glucose (H-2α) | 1.5-3.0 | 1.8-2.9 | 2.2-3.1 |

nd-not detectable.
